# Socioeconomic inequalities in neonatal and postneonatal mortality: Evidence from rural Iran, 1998–2013

**DOI:** 10.1186/s12939-017-0570-6

**Published:** 2017-05-18

**Authors:** Alireza Khajavi, Farhad Pishgar, Mina Dehghani, Shohreh Naderimagham

**Affiliations:** 1 Non-Communicable Diseases Research Center, Endocrinology and Metabolism Population Sciences Institute, Tehran University of Medical Sciences, Tehran, Iran; 2 Faculty of Paramedical Sciences, Shahid Beheshti University of Medical Sciences, Tehran, Iran; 3 Endocrinology and Metabolism Research Center, Endocrinology and Metabolism Clinical Sciences Institute, Tehran University of Medical Sciences, Tehran, Iran; 4 Students’ Scientific Research Center, Tehran University of Medical Sciences, Tehran, Iran; 5 Department of Pharmacoeconomics and Pharmaceutical Administration, School of Pharmacy, Tehran University of Medical Sciences, Tehran, Iran

**Keywords:** Healthcare disparities, Inequality, Infant mortality, Iran, Socioeconomic factors

## Abstract

**Background:**

Over the past three decades, interventions have been implemented to reduce childhood mortality in Iran. Despite declines in overall mortality rates, inequalities in mortality across socioeconomic groups have remained unchanged. In this study, we assessed inequalities in infant mortality in rural regions of Iran.

**Methods:**

We obtained data from the Iranian vital registration system, which includes data on 5,626,158 live births, 79,457 neonatal deaths, and 36,397 postneonatal deaths in rural areas of Iran over the course of a 16-year period, which was then divided into 4 four-year intervals. In addition to building multivariate regression models to identify factors associated with mortality, we calculated a concentration index for each province to measure inequalities in neonatal and postneonatal mortality, using wealth index as the socioeconomic variable of interest. We further assessed these inequalities as a component of their contributors by using the decomposition method.

**Results:**

Although both neonatal (17.62 to 10.92) and postneonatal (8.11 to 5.14) mortality rates exhibited decreasing trends from 1998-2001 to 2010–2013, the inequalities observed in these indices remained nearly unchanged (concentration indices of −0.062 to −0.047 and −0.098 to −0.083, respectively). Furthermore, fraction of births occurred in hospitals and literate women contributed positively to the inequalities observed in both neonatal and postneonatal mortality rates, whereas the proportion of infants classified as low birth weight contributed negatively over all study periods. We also identified decreasing trends in inequalities of the proportion of infants classified as having low birth weight, being born in hospitals, being covered by health insurance, mothers’ age, and literacy of women within the time intervals under study.

**Conclusions:**

Although infant mortality rates in Iran decreased over the studied time period, we observed notable inequalities in these measures. Several steps are needed to overcome these inequalities, including improving access to professional health services for lower income households, fairly distributing facilities and human resources, and improving insurance coverage to protect families from financial hardships. Moreover, social factors, such as literacy of women, were found to be important in decreasing inequalities in infant mortality. These steps require improving societal awareness of infant mortality and implementing improved and problem-oriented health policies.

## Background

Childhood mortality rates are among the most important health indices, representing the performance of societal health systems. The fourth Millennium Development Goal (Millennium Development Goal 4, MDG4) addresses child mortality, and governments and health authorities had been encouraged to work together to reduce the under-5 mortality rate (U5MR) by two thirds from 1990 to 2015 [1]. On the basis of global estimates, an overall annual reduction of 4.4% in U5MR was thought to be needed to achieve this goal. However, recent estimates have shown that reductions in U5MR were lower than expected, and on the global scale, an approximately 50% reduction in U5MR has been reported over the past 23 years, from a U5MR of 90 in 1990 to a U5MR of 46 in 2013, indicating an approximately 2.1% rate of reduction per year. Moreover, the predictions of the United Nations Interagency Group for Child Mortality (IGME) suggest that only 62 countries had reached the MDG4 by 2015 [2]. Differences in the reduction rates achieved among countries may be attributable to several factors, including financial factors, inhabitant education levels, implementation of public health programs and interventions, and access to new health technologies and systems [3–5].

Some health indices, such as child mortality, have shown strong associations with a country’s level of development. The more developed a country, the more likely it is to have lower child mortality rates, and higher child mortality rates are usually observed in less developed countries. In 2015, the MDGs were replaced by Sustainable Development Goals (SDGs), which also addressed the importance of childhood mortality [6]. The second target of the health-related SDGs promotes reducing the neonatal mortality rate (NMR) to 12 deaths per 1,000 live births and the U5MR to 25 deaths per 1,000 live births by 2030. An inevitable step in reaching these goals is to study past trends in NMRs and U5MRs and to identify factors that have either slowed the pace of childhood mortality reduction or have improved survival rates in children.

Studies have shown that a substantial fraction of mortality occurs during infancy, defined as the first year of life; worldwide, 70% of deaths among children under the age of 5 years have been found to occur in infants, a proportion that has increased steadily over the past several decades (from 67.4 in 1970 to 74.6% in 2015) [7]. The presence of heterogeneous mortality distributions across different age groups and different socioeconomic cohorts is not a new finding. However, assessing inequalities in the distribution of child mortality provides the opportunity to better understand past trends, investigate factors associated with the aforementioned health measures, and generate evidence-informed policies and interventions to reduce child mortality.

Few studies have investigated trends in child mortality inequalities in Iran, and most existing studies have evaluated these inequalities over short time periods or have been limited to subnational datasets. We conducted this nationally representative work to study inequalities in infant mortality in rural areas of Iran between 1998 and 2013, by using a previously described method [8]. Because the factors associated with child mortality may vary across different age groups, we categorized deaths occurring during infancy as neonatal, first month of life, and postneonatal mortality and studied them separately.

## Methods

### Data and variable definitions

The data used in this study were collected from two data sources. The first was the dataset from the Vital Horoscope (Zij) study, a registration system used for gathering annual demographic data in rural parts of Iran, including the numbers of births, low birth weight infants (weight less than 2.5 kilograms), hospital births, neonatal deaths, postneonatal deaths, and births, categorized into 5-year maternal age groups at the district level [9]. Moreover, the number of neonatal deaths per 1,000 live births (NMR), number of postneonatal deaths per 1,000 live births (PNMR), proportion of infants classified as low birth weight (LBW), proportion of in-hospital births (HB), and mean age of mothers (MAM) were calculated by using the aforementioned data.

The second data source was the Household Income and Expenditure Survey (HIES), which is disseminated annually by the Iranian Statistical Center. This dataset contains data for 1,175,364 individuals from 258,641 households. We used these data to extract information for four variables: the proportion of households covered by health insurance (INS), the proportion of households headed by men (HH), the proportion of literate women of reproductive age (ages of 15–49), and the average household wealth status (WI) in each district.

To measure household wealth status, a wealth index was defined by using the Principal Component Analysis (PCA) method to analyze participants’ answers to questions about the following 14 assets: home area; number of rooms; ownership of cars, televisions, refrigerators, ovens, vacuum cleaners, washing machines, media players, cell phones, and telephones; presence of in-home bathrooms and kitchens; and access to natural gas pipe lines. A wealth index was calculated by performing a PCA on these 14 assets, which contained 36% of the information available in these assets. Using the values calculated for each household, the mean wealth index of the households in each district was determined.

We further aggregated both datasets at the provincial level and then merged the two datasets and prepared them to analyze the effects of our variables of interest in 31 provinces over the course of 16 years.

### Analysis

Initial inspections of the data revealed similar trends in mortality rates and their six determinants in each consecutive 4-year study period. As a result, we divided the 16 years of study into 4 four-year intervals, and the analyses were performed over these periods. Furthermore, because deviations from the normal distribution were observed in the distribution of the evaluated mortality rates, a natural logarithm transformation was applied to facilitate the analysis.

To study factors associated with neonatal and postneonatal mortality rates, we built multivariate regression models by using the two mortality rates as response variables and six determinants (LBW, HB, MAM, INS, HH, and literacy) as explanatory variables for each of the 4 time periods of study. Additionally, in the regression models, the fixed effect of provinces were evaluated by using 30 dummy variables to compare each of the provinces with Tehran (as the reference province).

Further, for both of the two variables of interest (NMR and PNMR) in each of the 4-year time periods, we calculated a concentration index (CI) that measured inequality as a function of the distribution of a socioeconomic variable (wealth index in this study) [10]. The concentration index could be any value between −1 and +1, with zero indicating absolute equality, negative values indicating a higher rate of mortality in the groups of lower socioeconomic status, and positive values indicating a higher rate of mortality in the wealthier groups.

Next, we used the decomposing method, as previously described by Wagstaff et al., to investigate factors potentially associated with inequalities in child mortality rates [8]. Briefly, using the aforementioned regression models for our variables of interest (NMR and PNMR), shown as y in the following equation, we have:

1 $$ \mathrm{y}=\upalpha +{{\displaystyle {\sum}_{\mathrm{k}}\upbeta}}_{\mathrm{k}}{\mathrm{x}}_{\mathrm{k}}+\upvarepsilon $$

Coefficients for the 6 determinants (LBW, HB, MAM, INS, HH, and literacy) and 30 dummy variables corresponding to 31 provinces, denoted as x_k_, were calculated. Then, concentration indices for the variables of interest (y), denoted as C, were decomposed using the following equation:

2 $$ \mathrm{C}={\displaystyle \sum_{\mathrm{k}}}\left(\frac{\upbeta_{\mathrm{k}}{\overline{\mathrm{x}}}_{\mathrm{k}}}{\upmu}\right){\mathrm{C}}_{\mathrm{k}}+\frac{{\mathrm{GC}}_{\upvarepsilon}}{\upmu} $$

where ${\overline{\mathrm{x}}}_{\mathrm{k}}$ is the mean of x_k_, C_k_ is the concentration index for x_k_ in terms of the distribution of WI, μ is the mean of y, and GC_*ε*_ is the generalized concentration index for the error terms (ε). Then, ${\displaystyle \sum_{\mathrm{k}}}\left(\frac{\upbeta_{\mathrm{k}}{\overline{\mathrm{x}}}_{\mathrm{k}}}{\upmu}\right){\mathrm{C}}_{\mathrm{k}}$, the summation of concentration indices of all determinants, was calculated by applying ${\upbeta}_{\mathrm{k}}{\overline{\mathrm{x}}}_{\mathrm{k}}/\upmu$ as weights. The second component was defined as an indeterministic fraction of inequality that could not be explained. Hence, focusing on the first component of the equation,

3 $$ \widehat{\mathrm{C}}={\displaystyle {\sum}_{\mathrm{k}}}\left(\frac{\upbeta_{\mathrm{k}}{\overline{\mathrm{x}}}_{\mathrm{k}}}{\upmu}\right){\mathrm{C}}_{\mathrm{k}} $$

The contribution of each determinant to inequalities of mortality was calculated using the following equation:

$$ \left({\upbeta}_{\mathrm{k}}{\overline{\mathrm{x}}}_{\mathrm{k}}/\upmu \right){\mathrm{C}}_{\mathrm{k}}/\widehat{\mathrm{C}}*100. $$

*P*-values lower than 0.05 were considered to be statistically significant in this study. Statistical analyses were performed, and graphs were generated using Stata (version 9, Stata Corp, College Station, Texas, USA).

## Results

In rural areas in Iran, both neonatal and postneonatal mortality rates decreased over the study period. However, the proportions of infants classified as having low birth weight, being born in hospitals, and literacy of women of reproductive age showed steady increases. Similarly, mean maternal age increased over the study period; however, this increase was not as significant. Moreover, the rate of insurance coverage, especially after the second interval of the study period, showed a remarkable increase. Table 1 presents the average levels of the two variables of interest and six determinants in rural regions of Iran in each of the 4 time intervals under study.

**Table 1:** Average levels of neonatal and postneonatal mortality rates and the covariates

Variables	1998–2001		2002–2005		2006–2009		2010–2013	
	Mean	S.E	Mean	S.E	Mean	S.E	Mean	S.E
Neonatal mortality rate	17.62	0.19	15.84	0.17	13.12	0.16	10.92	0.13
Postneonatal mortality rate	8.11	0.13	6.87	0.11	5.99	0.09	5.14	0.08
Percent of births with low birth weight	4.25	0.05	4.81	0.05	5.40	0.06	5.98	0.08
Percent of births happened in hospital	79.15	0.61	88.63	0.42	95.36	0.25	98.07	0.14
Mean age of mothers	26.10	0.03	26.26	0.02	26.58	0.02	26.96	0.02
Percent of households covered with insurance	29.84	0.66	29.75	0.62	81.35	0.62	90.04	0.39
Percent of households with men as the head	94.63	0.14	94.67	0.13	93.25	0.14	91.76	0.15
Percent of literate women (at reproductive age, 15–49)	66.88	0.51	74.58	0.46	79.22	0.36	82.01	0.33

Neonatal and postneonatal mortality rates are shown as the number of reported deaths per 1,000 live births. The proportion of infants classified as having low birth weight, being born in hospitals, being covered by health insurance, households headed by men, and literate women of reproductive age are shown as percentages. S.E: standard error of the mean

Table 2 shows a summary of the proportion of births and neonatal and postneonatal mortality rates in the rural areas of each province in Iran over the years under study with the provinces ranked on the basis of wealth index over the 16-year period. Notably, the proportions of births in provinces of low socioeconomic status, such as Sistan & Baluchestan and Hormozgan, increased over the study period, whereas provinces of high socioeconomic status, such as Isfahan and Yazd, had decreased proportions of births over time.

**Table 2:** Proportion of births, neonatal and postneonatal mortality rates, and ranking of provinces on the basis of wealth index in rural regions of Iran

Provinces	1998–2001			2002–2005			2006–2009			2010–2013			Wealth Rank
	Proportion of births	NMR	PNMR	Proportion of births	NMR	PNMR	Proportion of births	NMR	PNMR	Proportion of births	NMR	PNMR	
Alborz	0.56	12.92	5.71	0.68	9.36	4.4	0.49	8.72	5.18	0.32	6.74	2.11	1
Tehran	1.34	10.53	4.38	1.54	7.05	3.38	1.99	4.43	2.63	1.06	3.79	2.84	2
Yazd	1.09	14.24	6.24	0.96	12.97	6.13	0.77	10.06	5.42	0.76	9.57	3.63	3
Mazandaran	5.32	13.63	4.67	5.33	10.87	3.66	4.79	8.91	3.46	4.77	7.09	2.71	4
Isfahan	4.36	17.63	6.19	3.43	15.18	5.32	2.83	11.36	4.94	2.55	8.93	3.44	5
Semnan	0.61	17.01	8.26	0.56	17.04	7.01	0.49	13.2	6.53	0.49	14.2	6.07	6
Bushehr	1.77	20.14	6.23	1.61	18.77	4.83	1.63	14.23	4.58	1.63	11.27	4.4	7
Qom	0.3	17.73	5.32	0.22	11.15	5.23	0.2	12.3	4.21	0.24	6.09	3.32	8
Qazvin	1.42	15.52	7.55	1.44	16.32	5.95	1.4	12.91	4.32	1.42	9.35	4.39	9
Golestan	4.08	18.16	9.59	4.38	17.49	7.02	5.43	11.64	5.54	5.37	9.62	4.52	10
Markazi	1.88	17.05	4.89	1.62	13.02	5.42	1.71	9.24	4.25	1.35	8.74	2.83	11
Fars	7.46	17.56	6.68	7.69	14.6	5.75	8.03	10.79	4.57	7.36	10.06	3.97	12
Gilan	5.09	16.18	4.35	4.37	15.72	3.7	3.07	12.69	3.47	2.8	10.48	2.46	13
Zanjan	1.7	18.23	8.29	2.28	16.71	5.99	1.97	15.11	5.26	1.97	9.46	4.01	14
Khuzestan	7.06	16.49	9.86	7.24	16.3	7.84	7.61	13.76	5.57	6.83	12.14	5.44	15
East Azarbaijan	5.67	17.71	7.59	5.7	13.77	6.63	5.19	10.44	5.2	5.45	6.96	4.62	16
Hamedan	3.77	17.61	8.04	3.61	17.02	6.1	3.28	12.1	4.65	3.42	12.26	3.93	17
West Azarbaijan	6.05	17.07	9.82	6.27	16.08	7.12	5.67	13.04	5.34	6.21	10.08	3.64	18
Chaharmahal & Bakhtiari	2.3	18.23	7.49	2.23	15.96	5.59	2.49	13.1	4.95	2.52	10.58	3.69	19
Ardebil	2.34	20.38	8.82	2.4	16.86	6.53	2.1	13.29	6.57	2.14	9.71	3.7	20
Ilam	0.86	17.87	6.69	0.94	12.93	5.45	1.12	12.1	4.78	0.82	11.48	4.35	21
Hormozgan	3.56	17.85	9.78	3.76	17.91	7.15	3.83	13.96	6.24	4.25	13.4	5.62	22
Razavi Khorasan	8.8	18.3	11.76	8.34	16.46	7.98	8.76	14.12	6.06	9.4	10.92	5.24	23
Kermanshah	2.63	22.41	8.03	2.68	17.51	5.73	2.42	15.06	5.07	2.46	13.85	4.79	24
North Khorasan	1.73	22.55	17.44	1.46	19.52	11.81	2.04	15.57	9.88	1.94	11.22	7.88	25
Kurdistan	2.91	27.15	9.16	2.94	22.62	5.55	2.63	16.07	4.76	2.54	11.84	4.03	26
Kerman	3.91	15.82	9.47	3.57	13.91	8.31	4.34	12.13	5.75	4.89	10.9	5.25	27
Kohgiluyeh and Boyerahmad	1.87	18.74	11.45	1.88	16.94	7.57	1.86	11.82	5.96	1.57	10.13	4.55	28
South Khorasan	1.45	21.91	15.24	1.2	20.49	10.79	1.17	17.74	8.76	1.35	14.45	7.08	29
Lorestan	2.99	21.09	7.78	3.23	20.47	6.3	3.17	12.84	5.22	3.44	10.51	4.07	30
Sistan & Baluchestan	5.12	18.61	19.14	6.45	18.47	13.62	7.53	15.09	10.52	8.69	12.79	8.27	31

Proportions of births are shown as percentages, whereas neonatal (NMR) and postneonatal (PNMR) mortality rates are presented as numbers of deaths per 1,000 live births. Wealth indices were used to sort the provinces on the basis of their wealth status

Table 3 shows results of the regression analyses performed to investigate the associations among several variables and neonatal and postneonatal mortality rates. Our results suggested that the proportion of infants classified as low birth weight was positively associated with neonatal mortality during each study period, and the proportions of in-hospital births and literate women of reproductive age were negatively associated with this mortality index over the time intervals under study. Furthermore, in the investigation of factors associated with the rate of postneonatal mortality, a positive association was identified between this index and mean maternal age, and negative associations were observed between the proportions of in-hospital births and literate women of reproductive age over the evaluated time intervals.

**Table 3:** Multivariate regression analysis of the associations of neonatal and postneonatal mortality rates in rural areas of Iran with the study variables

Study variables	Neonatal Mortality Rate				Postneonatal Mortality Rate			
	1998–2001	2002–2005	2006–2009	2010–2013	1998–2001	2002–2005	2006–2009	2010–2013
Proportion of births with low birth weight	**0.038**	**0.042**	**0.029**	**0.044**	0.012	0.017	0.012	0.009
Proportion of births happened in hospital	**−0.003**	**−0.007**	**−0.007**	−0.002	**−0.010**	**−0.011**	**−0.011**	**−0.024**
Mean age of mothers	−0.018	0.017	−0.005	−0.001	**0.052**	**0.056**	0.042	0.015
Proportion of households covered with insurances	0	0	−0.001	0	0.001	0.001	0	0
Proportion of households with men as the head	−0.002	0.001	0.006	−0.003	0	−0.005	−0.002	−0.003
Proportion of literate women (at reproductive age, 15–49)	**−0.004**	−0.001	−0.002	**−0.003**	−0.003	−0.003	**−0.006**	**−0.004**
Provinces								
Alborz	0.252	0.063	**0.311**	**0.738**	**0.420**	−0.026	0.392	−0.173
Ardebil	**0.536**	**0.642**	**0.805**	**0.717**	0.257	0.189	**0.338**	0.068
Bushehr	**0.519**	**0.776**	**0.846**	**0.916**	−0.028	−0.067	0.091	0.089
Chaharmahal & Bakhtiari	**0.377**	**0.538**	**0.901**	**0.853**	0.191	0.033	0.066	−0.092
East Azarbaijan	**0.465**	**0.470**	**0.541**	**0.400**	**0.258**	0.191	0.167	−0.010
Fars	**0.437**	**0.541**	**0.649**	**0.716**	0.098	0.037	0.182	−0.061
Gilan	**0.398**	**0.574**	**0.724**	**0.762**	**−0.245**	**−0.329**	−0.085	−0.147
Golestan	**0.395**	**0.621**	**0.635**	**0.637**	**0.299**	**0.267**	**0.301**	−0.044
Hamedan	**0.445**	**0.775**	**0.749**	**1.022**	**0.394**	0.222	0.077	−0.103
Hormozgan	**0.266**	**0.422**	**0.721**	**0.997**	0.037	−0.230	0.082	0.113
Ilam	**0.421**	**0.249**	**0.908**	**0.987**	0.045	0.015	0.224	0.064
Isfahan	**0.291**	**0.494**	**0.562**	**0.620**	0.209	0.032	**0.321**	0.165
Kerman	**0.182**	**0.367**	**0.724**	**0.822**	0.120	−0.095	0.178	0.145
Kermanshah	**0.640**	**0.756**	**0.875**	**1.163**	**0.296**	0.098	0.206	0.211
Khuzestan	**0.348**	**0.629**	**0.839**	**0.979**	**0.354**	0.233	0.195	0.215
Kohgiluyeh and Boyerahmad	**0.486**	**0.520**	**0.608**	**0.634**	0.177	−0.064	0.257	0.040
Kurdistan	**0.622**	**0.839**	**0.920**	**0.900**	0.139	−0.201	−0.129	−0.041
Lorestan	**0.552**	**0.848**	**0.693**	**0.841**	0.130	0.035	0.131	−0.167
Markazi	**0.277**	**0.442**	**0.521**	**0.590**	0.048	0.234	0.213	0.018
Mazandaran	**0.206**	**0.360**	**0.550**	**0.463**	−0.133	−0.233	−0.008	**−0.336**
North Khorasan	**0.568**	**0.710**	**0.902**	**0.894**	**0.658**	**0.468**	**0.455**	**0.374**
Qazvin	**0.329**	**0.642**	**0.597**	**0.563**	**0.336**	0.116	−0.017	0.030
Qom	**0.452**	0.318	**0.738**	0.293	−0.035	−0.084	0.028	−0.234
Razavi Khorasan	**0.401**	**0.583**	**0.787**	**0.803**	**0.485**	**0.261**	**0.240**	0.140
Semnan	**0.504**	**0.633**	**0.800**	**0.852**	**0.360**	0.332	**0.543**	0.205
Sistan & Baluchestan	0.084	**0.309**	**0.618**	**0.790**	0.275	0.253	0.249	0.083
South Khorasan	**0.572**	**0.435**	**0.990**	**0.912**	0.280	0.201	**0.414**	0.357
West Azarbaijan	**0.344**	**0.613**	**0.728**	**0.845**	0.039	0.036	−0.034	−0.283
Yazd	**0.196**	**0.349**	**0.504**	**0.676**	0.136	0.103	**0.491**	0.266
Zanjan	**0.371**	**0.597**	**0.822**	**0.584**	−0.187	0.127	0.223	0
**Adjusted R-Square (%)**	18.9	22.9	13.9	20.3	36.5	24.3	14.1	13.9

Regression models were generated to investigate the associations between neonatal and postneonatal mortality rates and the proportion of deliveries occurring in the hospital, mean maternal age, proportion of households covered by insurance, proportion of households headed by men, and proportion of literate women of reproductive age. Moreover, provincial effects were assessed using 30 dummy variables to compare the effect of each province, with Tehran as the reference group. The calculated coefficients are presented in the table and significant coefficients (those with *p*-values lower than 0.05) are shown in bold

### Concentration indices and their contributors

**Fig. 1:**
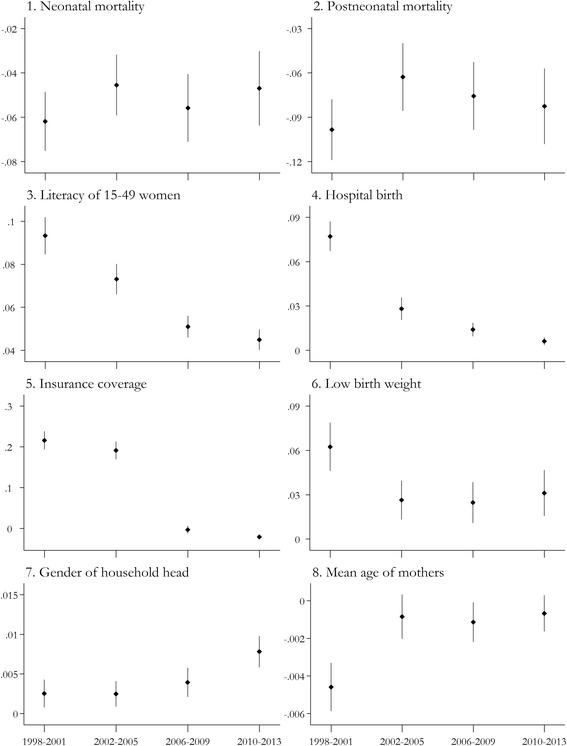
Concentration indices for neonatal and postneonatal mortality rates and their six associated factors, according to wealth index distribution. Concentration indices for neonatal and postneonatal mortality rates and the proportion of infants classified as having low birth weight, the proportion of deliveries occurring in hospitals, the mean maternal age, the proportion of households covered by any insurance, the proportion of households headed by men, and the proportion of literate women of reproductive ages are shown during the 4 time intervals under study. The levels of uncertainty for these indices are also presented in the graph. As mentioned previously, positive concentration index values indicated a positive correlation between wealth status and the variable of interest, whereas a negative concentration index value indicated a negative correlation. A concentration index of 0 indicated absolute equity in the distribution of study variable in respect to wealth status

The concentration indices for the two mortality rates and their six associated factors were calculated as a function of the distribution of wealth index over the four time periods under study. The calculated values, their confidence intervals, and changes in neonatal and postneonatal mortality inequalities are shown in Fig. 1. When inequalities were evaluated as a function of wealth status, the majority of the studied variables, including the proportion of infants classified as low birth weight, proportion of deliveries occurring in hospitals, mean maternal age, proportion of households covered by insurance, and proportion of literate women of reproductive age, decreased over the studied time periods. Inequalities in insurance coverage demonstrated an interesting trend. During the first two periods, the concentration indices had positive values of approximately 0.2; however, a significant decrease resulted in the concentration indices observed during the next two periods being approximately 0. However, inequalities in household head gender increased over the time intervals under study.

Using the previously described methods, the contributions of study variables and the effect of provinces were evaluated in association with inequalities in neonatal and postneonatal mortality over the time intervals of the study (Table 4). In our analysis, the proportion of infants classified as low birth weight was found to have a negative contribution to these rates, whereas the proportion of births occurring in hospitals and proportion of literate women had the strongest positive contributions to the inequalities observed in both mortality rates during each period. Moreover, regarding the determinants of postneonatal mortality, the proportion of households covered by insurance had a negative contribution and mean maternal age had a positive contribution during all periods under study.

**Table 4:** Contribution of different variables to the development of inequalities in neonatal and postneonatal mortality in rural areas of Iran between 1998 and 2013

Study variable	Neonatal Mortality Rate				Postneonatal Mortality Rate			
	1998–2001	2002–2005	2006–2009	2010–2013	1998–2001	2002–2005	2006–2009	2010–2013
Births with low birth weight	−6.671	−3.597	−2.114	−3.506	−2.206	−3.762	−1.964	−4.414
Births in hospital	10.867	12.138	4.792	0.640	45.605	47.819	18.478	40.499
Mean age of mothers	−1.474	0.258	−0.083	−0.008	4.516	2.149	1.586	0.758
Household with insurance coverage	1.853	0.697	−0.071	−0.215	−5.420	−9.686	−0.061	−0.649
Household with men as the household head	0.373	−0.200	−1.113	0.768	−0.066	2.000	0.817	6.736
Literate women at reproductive age, 15–49	15.228	4.408	3.984	4.873	13.069	26.202	32.235	40.395
Province								
Alborz	−0.025	−0.094	−0.162	0.035	−0.046	0.101	−0.466	−0.054
Ardebil	1.505	3.851	4.434	3.065	0.783	2.950	4.245	1.881
Bushehr	1.633	0.649	0.642	1.626	−0.097	−0.146	0.158	1.028
ChaharMahal & Bakhtiari	3.108	2.826	3.883	3.213	1.710	0.447	0.648	−2.267
East Azarbaijan	4.553	4.238	3.315	2.815	2.746	4.480	2.341	−0.452
Fars	4.011	3.177	4.272	4.178	0.972	0.571	2.740	−2.308
Ghazvin	1.543	1.133	0.944	0.978	1.714	0.530	−0.061	0.344
Gilan	4.458	4.259	3.585	3.292	−2.976	−6.330	−0.957	−4.129
Golestan	2.033	4.294	5.45	3.193	1.672	4.793	5.882	−1.441
Hamedan	5.904	9.076	5.138	6.191	5.673	6.742	1.209	−4.063
Hormozgan	1.950	1.448	3.019	7.142	0.294	−2.043	0.782	5.249
Ilam	1.354	0.794	2.451	1.310	0.158	0.125	1.380	0.556
Isfahan	0.735	0.215	−0.690	0.146	0.574	0.036	−0.901	0.252
Kerman	1.339	0.967	2.718	3.561	0.960	−0.647	1.520	4.083
Kermanshah	4.483	2.567	3.405	5.623	2.255	0.866	1.831	6.645
Khuzestan	3.489	3.913	6.914	7.225	3.856	3.768	3.666	10.357
Kohgilooye & Boyerahmad	3.478	2.487	3.027	2.914	1.376	−0.793	2.918	1.184
Kurdistan	6.664	6.892	5.407	4.795	1.615	−4.279	−1.724	−1.428
Lorestan	5.700	8.958	5.617	6.212	1.453	0.969	2.425	−8.039
Markazi	1.410	0.963	1.121	1.295	0.267	1.322	1.044	0.257
Mazandaran	0.713	−0.200	−0.986	0.305	−0.500	0.336	0.034	−1.440
North Khorasan	1.887	2.319	5.464	3.408	2.374	3.970	6.288	9.284
Qom	0.205	0.249	0.499	0.206	−0.017	−0.171	0.043	−1.069
Razavi Khorasan	8.483	6.609	7.047	5.669	11.154	7.695	4.902	6.458
Semnan	0.473	0.420	0.203	0.596	0.367	0.572	0.313	0.935
Sistan & Baluchestan	1.306	2.872	5.365	7.066	4.665	6.105	4.930	4.846
South Khorasan	2.223	0.273	1.655	1.838	1.182	0.328	1.578	4.691
West Azarbaijan	4.722	7.902	6.475	6.914	0.579	1.190	−0.699	−15.05
Yazd	0.005	−0.011	0.073	0.144	0.004	−0.009	0.162	0.369
Zanjan	0.480	3.250	4.322	2.491	−0.263	1.800	2.677	−0.002

Values are shown as percentages

## Discussion

In this paper, we investigated inequalities in the distribution of neonatal and postneonatal mortality in rural areas of Iran over the course of 16 years. Our findings showed that despite notable decreases in neonatal and postneonatal mortality rates over the study period, inequalities in distribution of these measures in Iran persisted, and higher neonatal and postneonatal mortality rates were still reported in areas of lower socioeconomic status.

The regression models built to evaluate neonatal mortality rates showed that the proportions of births occurring in hospitals and literate women of reproductive age were associated with lower mortality rates; additionally, higher neonatal mortality rates were observed in areas with a higher proportion of infants classified as having low birth weight. Moreover, in the evaluation of postneonatal mortality rates, the proportion of births occurring in hospitals and to younger mothers were associated with lower mortality rates.

We also evaluated inequalities in six determinants, including the proportion of infants classified as having low birth weight and being born in hospitals, the mean maternal age, the proportion of households with insurance coverage, the gender of household heads, and the proportion of literate women, in association with the average wealth status of rural areas. We found that in regions of higher socioeconomic status, greater proportions of births occurred in hospitals, more households were covered by insurance, and more women were literate. However, these inequality trends decreased during recent years. The values for inequality in insurance coverage dropped from 0.2 to approximately 0 after 2005, possibly as a consequence of the implementation of health system reform efforts in rural areas of Iran, known as the Family Physician Program and Social Protection Scheme for Rural Inhabitants. This program was implemented to provide rural inhabitants with insurance coverage, and it appears that this program has decreased inequalities in insurance coverage due to financial hardship [11, 12].

Furthermore, we evaluated inequalities in neonatal and postneonatal mortality indices and found that areas of lower socioeconomic status experienced significantly more infant deaths; however, a decreasing trend was observed in these rates. We also decomposed the inequalities observed in the evaluated mortality indices during each of the time periods and found that the proportions of hospitalized births and literate women contributed the most to the inequalities observed in both measures.

We also studied differences in mortality rates across different provinces and investigated the contribution of the province variable to the observed inequalities. Lower rates of neonatal and postneonatal mortality were identified in Tehran and Alborz, the provinces with the highest socioeconomic status, and the strong correlations were identified between higher rates of hospitalized births and lower rates of mortality highlighted the importance of access to facilities, such as neonatal intensive care unit (NICU) beds; receipt of care from skilled health workers; and financial resources in reducing childhood mortality.

The implications of the regression coefficients, concentration indices, and contribution of determinants to index inequality may be unclear. Two points may be helpful in this regard. First, in our work, a determinant had a positive contribution to inequality when its regression coefficient and concentration index exhibited opposite directionality. This characteristic indicated that a variable was associated with higher mortality rates, which were more frequently identified among people with lower socioeconomic status, and, therefore, indicated increased inequality in the distribution of the mortality index. The second point is that factors associated with infant mortality may not be similar to the determinants responsible for inequality in mortality distribution, although they might be interconnected [13]. For instance, in our work, the mean maternal age was a significant determinant of postneonatal mortality; however, its contribution to the inequality observed in this index was not notable.

Infant mortality rates and their associated factors have been studied previously, and because of the relationship between this index and socioeconomic factors, it is widely accepted that higher infant mortality rates are more prevalent among households with lower incomes or among mothers with lower educational levels. Moradi-Lakeh et al. have shown that geographical disparities in IMR and U5MR in Iran decreased from 1993 to 2008; however, the decreases in these disparities were not as substantial as the decreases observed in the indices themselves. The authors have suggested that providing patients with specialized care in addition to primary health services might improve this situation [5]. Our work showed similar results because hospitalized births, as an indicator of the availability of specialized health services and the patient wealth status, contributed substantially to inequalities in postneonatal mortality. In addition to the proportion of births that occurred in hospitals, the other factor that contributed the most to both inequalities in the rates of neonatal and postneonatal mortality in our study was the proportion of literate women. Our findings, once again, highlight the necessity of achieving a fair distribution of resources to provide patients with specialized health services and emphasize the roles of socioeconomic factors in reducing infant mortality.

Two previously published works have evaluated inequalities in infant mortality in Iran, both of which had used data from the 2000 Iranian Demographic and Health Survey (DHS). These data were collected via a national level survey administered in both urban and rural areas and include 108,875 live births and 3,908 infant deaths during the 10-year period from 1990 to 1999. Hosseinpoor et al. (2005 & 2006) have reported the concentration index for infant mortality to be −0.1789 and have suggested that household socioeconomic status, maternal education, birth interval, urban or rural residency, and hygienic status (access to toilets) contributed 36.2%, 20.9%, 13.0%, 13.9%, and 11.9% to the inequalities observed in infant mortality, respectively [14, 15]. In comparison, our study used data on infant deaths (divided to neonatal and postneonatal periods) occurring in rural areas over a longer time period, 1998 to 2013, which included 5,626,158 live births, 79,457 neonatal, and 36,397 postneonatal deaths, thus including a larger sample of subjects. Moreover, dividing deaths into neonatal and postneonatal groups and studying their associated factors separately is another difference between our work and that of Hosseinpoor et al. (2005 & 2006). Although we calculated lower concentration index values relative to those identified in their work, we also identified higher mortality rates among lower socioeconomic groups in this study.

Moreover, other studies on infant mortality rates in Iran have been conducted; these studies have used a descriptive approach or regression models to identify associations between infant mortality and socioeconomic variables. Movahedi et al. have reported that although infant and neonatal mortality decreased from 1993 to 2005, the inequalities observed in these measures remained unchanged. Although the authors have derived similar results, they relied on solely a visual inspection of maps to compare mortality rates between provinces [16]. Salarilak et al. have investigated factors associated with infant mortality in Iran and have reported that education of women, the socioeconomic status of households, and access to more specialized health services to be the main determinants of this mortality measure in Iran [17].

Although few works have investigated inequalities in the distribution of child mortality in Iran, the determinants of unequal distributions in this index have been previously studied in other parts of the world, including India, Pakistan, African countries, and Eastern European countries [18–23]. Among the factors cited as being associated with child mortality, household socioeconomic status, maternal education, health reform program implementation, a child’s birth order and the interval between births have been cited most frequently. Arif has assessed the factors associated with inequality in the distribution of child mortality in Pakistan between 2012 and 2013 [24]. His work has shown that child birth order, maternal education, and household socioeconomic status are associated with inequality in neonatal mortality, whereas maternal education, household socioeconomic status, and paternal education are determinants of inequality in postneonatal mortality. Vapattanawong et al. have shown a decreasing trend in child mortality in Thailand between 1990 and 2000, identifying a more prominent reduction in people with lower socioeconomic status [23]. These authors have attributed their results to interventions such as socioeconomic growth and a fair redistribution of the primary health care infrastructure. Similarly, a study of childhood health outcomes in Columbia supports the effect of primary health care implementation on reducing inequalities in infant mortality rates [25]. Although we found that the proportions of literate women, hospitalized births, and infants classified as having low birth weight contributed to the development of inequalities in infant mortality in rural areas of Iran, more comprehensive studies are required to investigate effects of other factors, such as birth intervals, paternal education, and health system reform, on the inequalities in these measures in Iran.

Our work has several limitations. First, the lack of a consistent registration system in urban areas limited our analysis to rural areas. However, previous studies have shown that inequalities in infant mortality rates are usually more prominent in rural areas [26, 27]. Second, we identified a significant association between wealth status and infant mortality at the provincial level in Iran; however, the observational nature of the study and the aggregated data used in our analyses limit this study to the identification of associations instead of cause-and-effect relationships**.**

## Conclusions

Here, we investigated the associations of several factors with and their contributions to inequalities in infant mortality, including lower levels of education among women, lower rates of hospitalized births, greater proportions of infants classified as low birth weight, mean material age, and lower socioeconomic status. Our work showed that factors such as the proportions of literate women and in-hospital births contributed to the development of inequalities in infant mortality rates across groups of different socioeconomic statuses.

In other words, although infant mortality rates decreased in Iran over the period under study, we report the presence of notable, albeit decreasing, inequalities in this measure. Further, we found that the proportions of literate women of reproductive age and the rate of in-hospital births explained a substantial portion of the inequalities observed in neonatal and postneonatal mortality across different socioeconomic groups. Several steps are needed to overcome these inequalities, including improved access to professional health services for households with lower incomes, which itself requires a fair distribution of facilities and human resources, as well as improved insurance coverage to protect families from financial hardships. Moreover, our work showed that social factors, such as literacy of women, are also important in decreasing inequalities in infant mortality. These steps require improving societal awareness of infant mortality and implementing revamped and problem-oriented health policies.
